# Health and health-related quality of life among treatment-seeking overweight and obese adults: associations with internalized weight bias

**DOI:** 10.1186/2050-2974-1-3

**Published:** 2013-01-22

**Authors:** Janet D Latner, Laura E Durso, Jonathan M Mond

**Affiliations:** 1 University of Hawai‘i at Manoa, 2350 Dole Street, Sakamaki C400, Honolulu, HI, 96822, USA; 2 The Williams Institute at UCLA School of Law, Box 951476, Los Angeles, CA, 90095, USA; 3 Centre for Rural and Remote Mental Health, School of Medicine and Public Health, University of Newcastle, Orange, NSW, 2800, Australia; 4 School of Sociology, Australian National University, Canberra, ACT, 0200, Australia

**Keywords:** Internalized weight bias, Health-related quality of life, Obesity, Overweight, Physical health impairment, Mental health impairment

## Abstract

**Background:**

Weight bias is widespread and has numerous harmful consequences. The internalization of weight bias has been associated with significant psychological impairment. Other forms of discrimination, such as racial and anti-gay bias, have been shown to be associated with physical health impairment. However, research has not yet examined whether internalized weight bias is associated with physical as well as psychological impairment in health-related quality of life.

**Methods:**

Participants included 120 treatment-seeking overweight and obese adults (mean body mass index = 35.09; mean age = 48.31; 68% female; 59% mixed or Asian ethnicity). Participants were administered measures of internalized weight bias and physical and mental health-related quality of life, and they were assessed for the presence of chronic medical conditions, use of prescription and non-prescription medications, and current exercise.

**Results:**

Internalized weight bias was significantly correlated with health impairment in both physical (r = −.25) and mental (r = −.48) domains. In multivariate analyses controlling for body mass index, age, and other physical health indicators, internalized weight bias significantly and independently predicted impairment in both physical (β = −.31) and mental (β = −.47) health.

**Conclusions:**

Internalized weight bias was associated with greater impairment in both the physical and mental domains of health-related quality of life. Internalized weight bias also contributed significantly to the variance in physical and mental health impairment over and above the contributions of BMI, age, and medical comorbidity. Consistent with the association between prejudice and physical health in other minority groups, these findings suggest a link between the effects of internalized weight-based discrimination and physical health. Research is needed on strategies to prevent weight bias and its internalization on both a societal and individual level.

## Background

Increased body weight is associated with impairments in health-related quality of life (HRQoL) [1]. Findings from population studies in different countries such as the U.S. [2,3], Sweden [4], Germany [5], and Taiwan [6] suggest that higher body mass index (BMI; kg/m^2^) is correlated with greater physical impairment in HRQoL. HRQoL, which refers to the effects of physical and mental health on everyday functioning, is increasingly regarded as a key outcome of health promotion programs and in clinical practice [7].

Overweight and obese individuals are far more likely to experience discrimination than their non-overweight peers [8]. This discrimination occurs in a range of settings, including medical, educational, and interpersonal contexts, and it is associated with a range of adverse personal, social, and economic outcomes [9,10]. One of the most pernicious aspects of weight-based discrimination is its tendency to become internalized. The term internalized weight bias (IWB), namely, the belief that weight-based stereotypes and prejudices are accurate and apply to the self, was introduced to describe this process [11]. In clinical and non-clinical obese samples, higher levels of IWB have been found to be associated with greater body dissatisfaction, more severe eating disturbances, higher levels of general psychological distress, and lower self-esteem [11-14].

In view of these associations, it is reasonable to hypothesize that IWB may be associated with impairment in HRQoL. Findings from one recent study suggested that IWB may be associated with poorer HRQoL among individuals with serious mental illness [15]. IWB was recently shown to mediate the relationship between BMI and weight-related quality of life, suggesting that weight-related impairments may be accounted for, in part, by weight-related self-stigma [16]. There is also evidence that discrimination is associated with adverse health outcomes in minority populations [17,18]. For example, perceived anti-gay discrimination predicted increased physician visits and medication use [19], while among African-American adolescents, perceived unfair treatment based on physical appearance was associated with elevated ambulatory blood pressure [20].

Research is needed to examine impairment in general HRQoL associated with IWB among individuals who are overweight or obese. In view of the multiple health-related conditions associated with obesity [21,22] and the impairment in HRQoL [1-6], it may be important to examine whether IWB predicts poorer physical and mental health outcomes. The goal of the present study was to address this gap in the literature by examining the associations between IWB and HRQoL in a treatment-seeking sample of overweight and obese individuals. We were particularly interested in considering whether any observed associations between obesity and impairment in HRQoL would still be apparent after controlling for the occurrence of those chronic medical conditions known to be associated with obesity and with physical and mental health impairment. We also wanted to test whether the health impairments associated with obesity are more closely associated with the psychological sequelae of being overweight in a stigmatizing society, than with body weight itself [23-25]. Therefore, the present cross-sectional study examined the association between IWB and HRQoL, while controlling for body mass index, as well as age and medical comorbidity.

## Methods

### Participants and procedures

Participants were individuals (n = 120) who were screened for a behavioral weight-loss treatment program, recruited from nine community centres across the Honolulu-metro area of Hawaii. Leaders of community centres who wished to participate in an obesity treatment program assisted the researchers in conducting recruitment among members via email, flyers, and announcements at group meetings. Centres included community groups such as the Young Men’s Christian Association, Young Women’s Christian Association, and religious institutions such as churches and temples. Participants interested in the treatment study attended an informational meeting at which they were screened for eligibility and completed the measures described below. The present study included all overweight individuals who applied and were screened to participate in the treatment trial, including those who were not eligible for participation (18%) or choose not to enroll in treatment (7%). Most individuals who were excluded from the trial were excluded on account of medical comorbidity but were included in the current study in order to maximize variability in the sample’s health status. Participants who were not included in the treatment trial did not differ from those who were included with respect to age, sex, or BMI. The study procedures were approved by the University of Hawaii Institutional Review Board, and informed consent was provided by all participants.

### Study measures

#### Weight bias internalization scale (WBIS)

The WBIS [4] assesses the degree to which respondents believe that negative stereotypes and negative self-statements about being overweight apply to themselves. Eleven questionnaire items are rated on a 7-point scale from “strongly disagree” to “strongly agree” (e.g. “As an overweight person, I feel that I am just as competent as anyone”). The WBIS has shown high internal consistency and convergent validity in a community sample of overweight and obese adults [11] as well as in a clinical sample of overweight and obese adults seeking treatment for binge eating disorder [26]. Higher scores indicate greater internalized bias. Internal consistency (Cronbach’s alpha) in the current sample was .71.

#### Medical outcomes study short-form (SF-12)

The SF-12 [27] is a widely used 12-item measure of health-related quality of life. Items are summarized into two weighted scales representing perceived impairment in role functioning associated with physical (Physical Component Summary scale, PCS) and mental (Mental Component Summary scale, MCS) health problems. The SF-12 has been demonstrated to have excellent reliability and validity [27]. Each scale is scored to have a mean of 50 and standard deviation of 10, with lower scores indicating greater impairment. The RAND scoring method, which employs factor weightings derived by means of oblique, rather than orthogonal, factor rotation, was used on account of its superior psychometric properties and recommended use in clinical samples [28]. Internal consistency (Cronbach’s alpha) in the current sample was .72 for the PCS and .76 for the MCS.

Information was also collected about the occurrence of chronic medical conditions and about current use of prescription and non-prescription medications. Participants filled out a checklist of 28 serious medical conditions (e.g., diabetes, hypertension), responded to on a *yes* or *no* basis. Medications currently taken were assessed by asking participants to rate the frequency with which they took eight categories of commonly used non-prescription medications over the past six months (rated on a Likert scale from 1 = *not at all* to 7 = *more than once a day*). They were also asked to indicate whether they were taking medications from a list of five categories of common prescription medications, responded to on a *yes* or *no* basis. Participants also indicated whether (*yes* or *no*) they were currently exercising regularly. Demographic information collected included age and ethnic background. BMI was calculated based on weight and height measured by a digital scale (Tanita BWBG-800) and stadiometer, respectively.

### Statistical analysis

Participant characteristics were summarized by computing HRQoL subscale scores, WBIS scores, and health-related variables. Analysis of covariance controlling for BMI and age was used to explore potential differences in WBIS scores between participants who reported the presence or absence of a serious medical disorder, use or non-use of prescription medication, and the occurrence or absence of regular exercise. To assess the relationship between internalized bias and quality of life, Pearson product moment correlations were computed between WBIS scores and PCS and MCS. Hierarchical multiple regression analysis was used to examine the independent contribution of WBIS scores to the variance in PCS and MCS scores, controlling for BMI, age, and physical health indicators. Health indicators entered included the presence or absence of any serious medical condition, frequency of non-prescription medication use, and the occurrence or absence of regular exercise (dichotomous variables were dummy coded). By entering the variables of BMI, age, exercise, medical conditions, and medication use in step one, and IWB in step 2, this analysis permitted the identification of the independent contribution of IWB over and above the step 1 variables. In preliminary analyses stratified by gender, effect sizes were similar in women and men; therefore, data were combined in subsequent analyses.

## Results

### Participant characteristics

Participants had a mean BMI of 35.09 (SD = 7.65) and mean age of 48.31 (SD = 13.32), and 67.5% were female. The sample was 29% overweight and 71% obese (BMI range: 25.2 – 66.4; age range: 20–77 years). Participants self-identified as Caucasian (30%), Asian (20%), Pacific Islander or Native Hawaiian (8%), Latino or Latina (3%), and mixed ethnicity (39%). Sixty-seven percent of participants reported the presence of a serious medical condition, 58% reported currently taking a prescription medication, and 15% reported currently exercising. Across eight categories of non-prescription drugs, the mean frequency of reported use was 1.77 (SD = .76), indicating that, on average, participants had taken several non-prescription medications “*once or twice*” in the past six months. After controlling for BMI and age, there were no differences in WBIS scores between participants who reported the presence or absence of a serious medical condition, use or non-use of prescription medication, and the occurrence or absence of regular exercise. Mean scores (SD) on WBIS, PCS, and MCS in the total sample were, respectively, 3.86 (1.06), 45.78 (8.62), and 45.47 (9.20).

### Associations between IWB and HRQoL

WBIS scores were significantly correlated with both the physical health (r = −.25, p = .01) and mental health (r = −.48, p < .001) subscales of the SF-12. These correlations indicate effect sizes of a medium and large magnitude, respectively [29]. The results were unchanged when the effects of BMI were controlled by means of partial correlation analysis.

### Relative contribution of IWB to HRQoL

Separate hierarchical regression analyses were conducted for SF-12 PCS and MCS scores with BMI, age, and physical health indicators (exercise, medical conditions, and medication use) entered at the first step and WBIS scores entered at the second step. As shown in Table 1, WBIS scores significantly predicted PCS scores, over and above the contributions of BMI, age, and physical health indicators. Higher WBIS scores were associated with lower PCS scores (greater physical health impairment). The occurrence of regular exercise was also independently associated with PCS scores, such that participants who exercised regularly had higher PCS scores than those who did not.

**Table 1:** Hierarchical multiple regression analyses predicting physical health-related quality of life and mental health-related quality of life

		**Physical component summary**		
**Predictor variables**	**Δ R**^**2**^	**β**
Step 1	.18^a^	
BMI		-.06
Age		-.01
Exercise		.38^b^
Medical condition		-.07
Medication use		-.05
Step 2	.09^a^	
WBIS		-.31^b^
Total R^2^	.26^b^	
**Mental component summary**		
**Predictor variables**	**Δ R**^**2**^	**β**
Step 1	.05	
BMI		.08
Age		.22
Exercise		.16
Medical condition		-.04
Medication use		-.01
Step 2	.21^b^	
WBIS		-.47^b^
Total R^2^	.26^b^	

Note. ^a^p < .01, ^b^p < .001.

A similar pattern was observed when the SF-12 MCS was the dependent variable. Higher scores on the WBIS were associated with lower MCS scores (greater mental health impairment) over and above the contributions of BMI, age, and physical health indicators. In this case, WBIS was the only significant predictor variable.

## Discussion

We examined associations between IWB and impairment in HRQoL in a treatment-seeking sample of overweight and obese men and women. The main findings were twofold. First, IWB was associated with greater perceived impairment in both the physical and mental health domains of HRQoL. Second, IWB contributed significantly and independently to the variance in physical and mental health impairment over and above the contributions of BMI, age, exercise, medical conditions, and medication use. The association between IWB and health impairment among overweight and obese individuals is consistent with past studies demonstrating that prejudice and internalized bias may be linked to adverse health outcomes in other commonly stigmatized groups, such as gays, lesbians, bisexuals, and ethnic minorities [18,30,31]. For example, internalized racism has been associated with negative health outcomes among African Americans [32,33]. It has been proposed that the stress associated with IWB may have a significant impact on cardiovascular health and metabolic abnormalities, which could potentially lead to poorer health, weight gain, and increased risk of internalized stigma [10]. Stress associated with internalized bias may also lead to unhealthy coping behaviors, such as smoking, alcohol or substance use [34] or binge eating [12]. Another potential coping behavior that could lead to poor HRQoL is avoidance. Phobic anxiety and avoidance of specific situations may result among obese individuals who have experienced weight stigma [35].

Although the cross-sectional design of the current study precludes any conclusions concerning the direction of the observed associations, it is worth considering the possibility that weight bias and its internalization may be conducive to adverse health outcomes among overweight and obese individuals, that is, in addition to the more commonly accepted view that impairment in HRQoL is a consequence of obesity. The present findings suggest that future research using prospective study designs should continue to address this issue.

The finding that, in this clinical sample of overweight and obese individuals, IWB was associated with significant impairment in both physical and mental health domains of HRQoL, independent of its association with medical comorbidity, suggests that it may be important to include the reduction of IWB as a key therapeutic goal of obesity treatment programs. There is some evidence that this goal is viable. For example, a day-long intervention directed at alleviating internalized bias by teaching acceptance and mindfulness significantly reduced IWB in obese participants in one recent trial [36]. Future research of this kind should include assessment of the potential benefits of reducing IWB on specific aspects of HRQoL. Whether weight bias can be reduced at the population level is less clear [37]. Therefore, from a public health perspective, it may be especially important to test the efficacy of health promotion and early intervention programs that seek to preempt the adverse effects of IWB on obese individuals’ quality of life.

Other limitations of the present study - that is, other than the use of cross-sectional study design - should be noted. First, we relied on a single, brief measure of HRQoL that yields scores on subscales relating to only physical and mental health impairment. Although the SF-12 is widely used and has good psychometric properties, the use of a more comprehensive measure, such as the SF-36, would be desirable. It would also be desirable to include an obesity-specific measure of HRQoL in future research e.g., [38], particularly in research designed to evaluate the effects of clinical intervention on levels of IWB, since a measure of this kind may be more sensitive to change. It would be interesting to examine how strong the correlation might be between IWB and an obesity-specific measure of HRQoL. Second, and whereas the assessment of medical comorbidity is a strength of the present study, other variables likely to be associated with both body weight and HRQoL were not assessed. The role of body dissatisfaction and eating-disordered behavior may be particularly important in this regard [23,39,40]. Future studies should also examine the relative effects of depression alongside those of IWB. Considering the relationship between obesity, depression, and HRQoL [41], it is possible that depression may account for variance in HRQoL or may mediate the relationship between WBIS and HRQoL. In addition, it should be noted that the assessment of medical comorbidity and related variables was by self-report. Data bearing on actual (diagnosed) medical comorbidity and documented use of medication would, of course, be preferable. Finally, the current study included a large proportion of Asian participants and participants of mixed (primarily Asian, Pacific Islander, and Caucasian) descent. The use of this this study population may limit the generalizability of the findings, although the choice of study population might also be considered a strength of the study, given the reliance on Western populations in most previous research. In any case, further research exploring the associations of IWB with impairment in quality of life, in different study populations, would be welcome.

## Conclusions

In sum, the current findings suggest that internalized bias may be associated not only with important psychological outcomes, as shown in past research [11-14,26], but also with physical health outcomes as well. Given the relative ineffectiveness of studies attempting to reduce obesity stigma at a societal level [37], it may be important to focus on preventing the potential negative psychological outcomes associated with IWB among overweight and obese patients. It is possible that targeting IWB in clinical settings might improve not only patients’ psychological well-being, but perhaps their physical health functioning as well.

## Competing interests

The authors declare that they have no competing interests.

## Authors’ contributions

JDL obtained the grant from which this research was funded, conceived of the study design, carried out analyses, and drafted the manuscript. LED participated in the design of the study, contributed to the statistical analyses, and helped edit the manuscript. JMM participated in the design of the study and helped to edit the manuscript. All authors read and approved the final manuscript.

## References

[B1] de Zwaan M Petersen I Kaerber M Burgmer R Nolting B Legenbauer T Benecke A Herpertz S Obesity and quality of life: a controlled study of normal-weight and obese individuals Psychosomatics 2009 50 474 482 19855033 10.1176/appi.psy.50.5.474

[B2] Jia H Lubetkin EI The impact of obesity on health-related quality-of-life in the general adult US population J Public Health 2005 27 156 164 10.1093/pubmed/fdi025 15820993

[B3] Hassan MK Joshi AV Madhavan SS Amonkar MM Obesity and health-related quality of life: a cross-sectional analysis of the US population Int J Obes 2003 27 1227 1232 10.1038/sj.ijo.0802396 14513071

[B4] Larsson U Karlsson J Sullivan M Impact of overweight and obesity on health-related quality of life – a Swedish population study Int J Obes 2002 26 417 424 10.1038/sj.ijo.0801919 11896499

[B5] Mond JM Baune BT Overweight, medical comorbidity and health-related quality of life in a community sample of women and men Obesity 2009 17 1627 1634 10.1038/oby.2009.27 19629059

[B6] Huang I-C Frangakis C Wu AW The relationship of excess body weight and health-related quality of life: evidence from a population study in Taiwan Int J Obes 2006 30 1250 1259 10.1038/sj.ijo.0803250 16520814

[B7] Calvert M Blazeby J Revicki D Moher D Brundage M Reporting quality of life in clinical trials: a CONSORT extension Lancet 2011 378 1684 1685 10.1016/S0140-6736(11)61256-7 22078674

[B8] Carr D Friedman MA Is obesity stigmatizing? body weight, perceived discrimination, and psychological well-being in the united states J Health Soc Beh 2005 46 244 259 10.1177/002214650504600303 16259147

[B9] Puhl R Heuer CA The stigma of obesity: a review and update Obesity 2009 17 941 964 10.1038/oby.2008.636 19165161

[B10] Puhl RM Latner JD Stigma, obesity, and the health of the nation’s children Psychol Bulletin 2007 133 557 580 10.1037/0033-2909.133.4.557 17592956

[B11] Durso LE Latner JD Understanding self-directed stigma: development of the weight bias internalization scale Obesity 2008 16 S80 S86 18978768 10.1038/oby.2008.448

[B12] Durso LE Latner JD Hayashi K Discrimination as a risk factor for binge eating in stigmatized groups Obes Factsin press 10.1159/000345931 23258192

[B13] Carels RA Wott CB Young KM Gumble A Koball A Oehlhof MW Implicit, explicit and internalized weight bias and psychosocial maladjustment among treatment-seeking adults Eat Behav 2010 11 180 185 10.1016/j.eatbeh.2010.03.002 20434066

[B14] Roberto CA Sysko R Bush J Pearl R Puhl RM Schvey NA Dovidio JF Clinical correlates of the weight bias internalization scale in a sample of obese adolescents seeking bariatric surgery Obesity 2012 20 533 9 10.1038/oby.2011.123 21593805 PMC3481186

[B15] Barber JA Palmese L Reutenauer EL Grilo CM Tek C Implications of weight-based stigma and self-bias on quality of life among individuals with schizophrenia J Nerv Ment Dis 2011 199 431 435 10.1097/NMD.0b013e318221403d 21716053 PMC3135000

[B16] Lillis J Levin ME Hayes SC Exploring the relationship between body mass index and health-related quality of life: a pilot study of the impact of weight self-stigma and experiential avoidance J Health Psychol 2011 16 722 727 21441358 10.1177/1359105310388321

[B17] Krieger M Embodying inequality: a review of concepts, measures and methods for study Int J Health Services 1999 29 295 352 10.2190/M11W-VWXE-KQM9-G97Q 10379455

[B18] Pascoe EA Richman LS Perceived discrimination and health: a meta-analytic review Psychol Bulletin 2009 135 531 554 10.1037/a0016059 PMC2747726 19586161

[B19] Huebner DM Davis MC Perceived anti-gay discrimination and physical health outcomes Heal Psychol 2007 26 627 634 10.1037/0278-6133.26.5.627 17845114

[B20] Matthews KA Salomon K Kenyon K Zhou F Unfair treatment, discrimination, and ambulatory blood pressure in black and white adolescents Heal Psychol 2005 24 258 265 10.1037/0278-6133.24.3.258 15898861

[B21] Bray GA Medical consequences of obesity J Clin Endocrinol Metab 2004 89 2583 2589 10.1210/jc.2004-0535 15181027

[B22] Malnick SDH Knobler H The medical complications of obesity Q J Med 2006 99 565 579 10.1093/qjmed/hcl085 16916862

[B23] Mond JM Rodgers B Hay PJ Darby A Owen C Baune BT Kennedy RL Obesity and impairment in psychosocial functioning in women: the mediating role of eating disorder features Obesity 2007 15 2769 2779 10.1038/oby.2007.329 18070768

[B24] Muennig P Jia H Lee R Lubetkin E I think therefore I am: perceived ideal weight as a determinant of health Am J Public Health 2008 98 501 506 10.2105/AJPH.2007.114769 18235062 PMC2253567

[B25] Muennig P Bench KK Obesity-associated stigma and physiological markers of stress: evidence from the Dominican republic Stress Heal 2009 25 241 246 10.1002/smi.1243

[B26] Durso LE Latner JD White MA Masheb RM Blomquist KK Morgan PT Grilo CM Internalized weight bias in obese patients with binge eating disorder: associations with eating disturbances and psychological functioning Int J Eat Disord 2012 45 423 427 10.1002/eat.20933 21717488 PMC3184343

[B27] Ware J Kosinski M Keller SD A 12-item short-form health survey: construction of scales and preliminary tests of reliability and validity Med Care 1996 34 220 33 10.1097/00005650-199603000-00003 8628042

[B28] Windsor TD Rodgers B Butterworth P Anstey KJ Jorm AF Measuring physical and mental health using the SF-12: implications for community surveys of mental health Aust NZ J Psych 2006 40 797 803 10.1080/j.1440-1614.2006.01886.x 16911756

[B29] Cohen J A power primer Psychol Bull 1992 112 155 159 19565683 10.1037//0033-2909.112.1.155

[B30] Institute of Medicine The health of lesbian, Gay, bisexual, and transgender people: building a foundation for better understanding 2011 Washington, DC: The National Academies Press 22013611

[B31] Meyer IH Prejudice, social stress, and mental health in lesbian, gay, and bisexual populations: Conceptual issues and research evidence Psychol Bulletin 2003 129 674 697 10.1037/0033-2909.129.5.674 PMC2072932 12956539

[B32] Butler C Tull ES Chambers EC Taylor J Internalized racism, body fat distribution, and abnormal fasting glucose among African-Caribbean women in Dominica, West Indies J Nat Med Assoc 2001 94 143 148 PMC2594102 11918383

[B33] Tull ES Sheu YT Butler C Cornelious K Relationships between perceived stress, coping behavior and cortisol secretion in women with high and low levels of internalized racism J Nat Med Assoc 2005 97 206 212 PMC2568780 15712783

[B34] Cooper H Friedman S Tempalski B Friedman R Keem M Racial/ethnic disparities in injection drug use in large US metropolitan areas Ann Epidemiology 2005 15 326 334 10.1016/j.annepidem.2004.10.008 15840545

[B35] Friedman KE Ashmore JA Applegate CL Recent experiences of weight-based stigmatization in a weight loss surgery population: Psychological and behavioral correlates Obesity 2008 16 S69 S74 18978766 10.1038/oby.2008.457

[B36] Lillis J Luoma JB Levin ME Hayes SC Measuring weight self-stigma: The weight self-stigma questionnaire Obesity 2010 18 971 976 10.1038/oby.2009.353 19834462

[B37] Daníelsdóttir S O’Brien KS Ciao A Anti-fat prejudice reduction: A review of published studies Obes Facts 2010 3 47 58 10.1159/000277067 20215795 PMC6452150

[B38] Kolotkin RL Crosby RD Kosloski KD Williams GR Development of a brief measure to assess quality of life in obesity Obes Res 2001 9 102 111 10.1038/oby.2001.13 11316344

[B39] Mond JM van den Berg P Boutelle K Neumark-Sztainer D Hannan PJ Obesity, body dissatisfaction, and psycho-social functioning in early and late adolescence: findings from the Project EAT Study J Adolescent Health 2011 48 373 378 10.1016/j.jadohealth.2010.07.022 PMC3214691 21402266

[B40] Hay P Mond J Buttner P Darby A Eating disorder behaviors are increasing: Findings from two sequential community surveys in South Australia PLOSone 2008 3 e1541 10.1371/journal.pone.0001541 PMC2212110 18253489

[B41] Vetter ML Wadden TA Lavenberg J Moore RH Volger S Perez JL Sarwer DB Tsai AG Relation of health-related quality of life to metabolic syndrome, obesity, depression and comorbid illnesses Int J Obes 2011 35 1087 1094 10.1038/ijo.2010.230 PMC3085045 21042326

